# Antiviral Activity of Zinc Oxide Nanoparticles and Tetrapods Against the Hepatitis E and Hepatitis C Viruses

**DOI:** 10.3389/fmicb.2022.881595

**Published:** 2022-06-23

**Authors:** Jyoti Gupta, Minnah Irfan, Niranjan Ramgir, K. P. Muthe, A. K. Debnath, Shabnam Ansari, Jaya Gandhi, C. T. Ranjith-Kumar, Milan Surjit

**Affiliations:** ^1^Virology Laboratory, Vaccine and Infectious Disease Research Centre, Translational Health Science and Technology Institute, NCR Biotech Science Cluster, Faridabad, India; ^2^University School of Biotechnology, Guru Gobind Singh Indraprastha University, New Delhi, India; ^3^Technical Physics Division, Bhabha Atomic Research Center, Mumbai, India

**Keywords:** hepatitis E virus (HEV), zinc oxide nanoparticles, zinc oxide tetrapod, antivirals, hepatitis C virus (HCV)

## Abstract

Hepatitis E virus (HEV) causes an acute, self-limiting hepatitis. The disease takes a severe form in pregnant women, leading to around 30% mortality. Zinc is an essential micronutrient that plays a crucial role in multiple cellular processes. Our earlier findings demonstrated the antiviral activity of zinc salts against HEV infection. Zinc oxide (ZnO) and its nanostructures have attracted marked interest due to their unique characteristics. Here we synthesized ZnO nanoparticles [ZnO(NP)] and tetrapod-shaped ZnO nanoparticles [ZnO(TP)] and evaluated their antiviral activity. Both ZnO(NP) and ZnO(TP) displayed potent antiviral activity against hepatitis E and hepatitis C viruses, with the latter being more effective. Measurement of cell viability and intracellular reactive oxygen species levels revealed that both ZnO(NP) and ZnO(TP) are noncytotoxic to the cells even at significantly higher doses, compared to a conventional zinc salt (ZnSO_4_). Our study paves the way for evaluation of the potential therapeutic benefit of ZnO(TP) against HEV and HCV.

## Introduction

Zinc is an essential trace element, which is naturally present in many food items. It is necessary for growth, development, and maintenance of immune function in human beings. A healthy adult body contains ~1.5–2.0 g of zinc. It is also an essential component of many enzymes and zinc finger motif-containing transcription factors (Maret and Sandstead, 2006; John et al., 2010). Zinc deficiency leads to a wide range of disorders, including metabolic diseases, compromised immune system, developmental imbalances, neurodegenerative problems, and inflammatory pathologies (Simmer and Thompson, 1985; Fabris and Mocchegiani, 1995; Prasad, 2008). Importantly, it is known to possess antiviral properties against many different viruses, namely, herpes simplex virus (HSV), severe acute respiratory syndrome coronavirus (SARS CoV), rhinovirus, respiratory syncytial virus (RSV), equine arteritis virus (EAV), human papilloma virus (HPV), human immunodeficiency virus (HIV), hepatitis C virus (HCV), and hepatitis E virus (HEV) (Korant et al., 1974; Haraguchi et al., 1999; Suara and Crowe Jr, 2004; Te Velthuis et al., 2010; Kaushik et al., 2017). Multiple mechanisms underlie the antiviral activity of zinc, which includes inhibition of virus entry and viral polyprotein processing or inhibition of viral RNA dependent RNA polymerase activity (Haraguchi et al., 1999; Krenn et al., 2009; Te Velthuis et al., 2010; Kaushik et al., 2017). Zinc also contributes toward modulating the host immune response to restrict viral replication. It is a mediator of LPS (bacterial lipopolysaccharides)-induced TLR4 (Toll-like receptor 4)-dependent MyD88 (myeloid differentiation primary response protein 88) signaling pathway that leads to early activation of NF-κB (nuclear factor-kappa B). This subsequently activates the production of pro-inflammatory cytokines such as TNFα (tumor necrosis factor α), IL-1β (interleukin-1β), and IL-6 (interleukin-6). These cytokines play important roles in controlling the viral pathogen (Haase et al., 2008; Brieger et al., 2013).

Despite its proven antiviral activity *in vitro*, the control of viral diseases by zinc supplementation therapy is not easy, mainly due to the tight control of zinc homeostasis *in vivo*. Zinc is usually stored in intracellular vesicles to ensure that the level of intracellular free zinc is tightly controlled. A number of zinc transporters mobilize zinc from the intracellular stores and from the systemic circulation depending on the requirement, a process predominantly regulated by the glucocorticoid, insulin, and glucagon hormones (Prasad, 1995; Kambe et al., 2015; Olechnowicz et al., 2018). Therefore, administration of excess zinc does not always result in a corresponding increase in the level of intracellular free zinc. On average, the body absorbs only 10–20% of the ingested zinc. Excess zinc leads to cytotoxicity, thereby restricting the therapeutic benefit of exogenously administered zinc (Hamatake et al., 2000; Plum et al., 2010).

Zinc oxide (ZnO) has a wide spectrum of biological properties, and accordingly, it has been used in various commercial pharmaceutical products. It has also been used against bacterial diseases and skin cancer (Akhtar et al., 2012; Jiang et al., 2018). Variants of ZnO have been reported to improve its utility. For example, a zinc oxide nanoparticle ZnO(NP) formulation has been shown to be better absorbed in the intestine and possesses better bioavailability and reduced undesirable side effect characteristics (Sirelkhatim et al., 2015; Jiang et al., 2018). Recently, another variant of ZnO, namely, the tetrapod-shaped ZnO [ZnO(TP)] has been reported to prevent herpes simplex virus (HSV) infection. It has adjuvant like properties, enhances viral presentation to the dendritic cells, and increases cell-mediated and humoral immunity (Antoine et al., 2012, 2016). Antiviral effects of ZnO(NP) have been tested against influenza, HSV1, and HSV2 (Mishra et al., 2011; Antoine et al., 2012, 2016; Ghaffari et al., 2019). This has been attributed mainly to the immune modulating potential of the ZnO nanoparticles. The nanoparticles activate the innate and adaptive immune response by triggering the Toll-like receptor signaling pathways and their downstream proteins, which leads to the secretion of pro-inflammatory cytokines that restrict the virus.

Viral hepatitis is a major human health concern. For instance, hepatitis E virus (HEV) infection causes ~50% of acute viral hepatitis in India. It also accounts for a significant number of acute liver failure (ALF) and acute-on-chronic liver failure (ACLF) (Wu et al., 2021). Extra hepatic manifestations of HEV infection have also been reported (Bazerbachi et al., 2016; Fousekis et al., 2020). HEV is a single-stranded, positive-sense RNA virus belonging to the family Hepeviridae (Smith et al., 2014). It is mainly transmitted through the gastrointestinal route by contaminated water or food of animal origin. The virus that infects mammals is classified into seven genotypes and one serotype. Out of the seven, six genotypes are known to infect humans (Smith et al., 2014, 2020). Genotypes 1 and 2 viruses cause acute hepatitis in the general population, with a case fatality rate of 0.5–3% and up to 30% of fatality has been reported in pregnant women infected with genotype 1-HEV. Genotype 3-HEV is the main cause of chronic infection in elderly or immunocompromised patients, such as organ transplant recipients and HIV-infected individuals, and they have a high risk of progressing to liver cirrhosis (Kamar et al., 2008; Dalton et al., 2009; Ollier et al., 2009; Nan and Zhang, 2016). Sporadic cases of chronic infection by genotype 4-HEV are also reported. Human infection by genotype 7-HEV and genotype 8-HEV has been reported (Smith et al., 2020). Currently, there is no HEV-specific therapeutic protocol. A combination of ribavirin and pegylated IFN is used as an off-label treatment option for chronic hepatitis E (Kamar et al., 2010a,b, 2014).

Hepatitis C virus (HCV) is an enveloped, positive-sense, single-stranded RNA virus belonging to the family Flaviviridae. Its infection is one of the leading causes of chronic liver disease, hepatocellular carcinoma, and the major indicator for liver transplantation in Western countries (Liang et al., 2000; Fattovich et al., 2004; Thomas, 2013). There are seven major genotypes of HCV that lead to differential prognosis of hepatitis C disease and influence antiviral therapy selection (Gower et al., 2014; Saraswat et al., 2015). Genotype 3-HCV is a major cause of liver cirrhosis and hepatocellular carcinoma in developing countries (Gondeau et al., 2015).

Earlier studies in our laboratory have demonstrated the dose-dependent inhibitory effect of zinc salts on HEV replication (Kaushik et al., 2017). In this study, the anti-viral effect of ZnO(NP) and ZnO(TP) against HEV was evaluated and compared to that of zinc sulfate (ZnSO_4_) and a few other known inhibitors of HEV replication. Additionally, the antiviral effects of ZnO(NP) and ZnO(TP) were investigated using HCV replicon and compared the results to those of ZnSO_4_ and sofosbuvir, a known antiviral against HCV.

## Materials and Methods

### Mammalian Cell Culture

Human hepatoma (Huh7) cells were originally obtained from Professor Charles M. Rice and are maintained in our laboratory (Nair et al., 2016). Cells were revived from liquid nitrogen stock and maintained in Dulbecco's modified Eagle medium (DMEM) containing 10% fetal calf serum (FCS) and 50 IU/ml penicillin and streptomycin in a humidified incubator at 37°C supplied with 5% CO_**2**_. An antibiotic was not added to the culture medium while seeding the cells for the experiment. Different zinc compounds and other antivirals were directly added to the culture medium at the indicated final concentration. Unless specified otherwise, all treatments were done once, followed by incubation for 24 h.

### Cell Viability Assay

To measure the cytotoxic effect of ZnO(NP), ZnO(TP), and ZnSO_4_, a cell viability assay was performed using a CellTiter 96 AQueous One Solution Cell Proliferation Assay kit (Promega, USA), following the manufacturer's instructions. Briefly, 10^4^ Huh7 cells were seeded into a 96-well plate and incubated at 37°C in a humidified 5% CO_2_ incubator. The following day, the media was replaced with fresh media and cells were treated for 24 h with an increased concentration of ZnO(NP), ZnO(TP), and ZnSO_4_, starting from 100 μM to 1 mM. After 24 h, 20 μl of MTS solution was added to each well, and the plate was incubated at 37°C for 4 h in the incubator, followed by measurement of absorbance at 490 nm.

### Reactive Oxygen Species (ROS) Estimation

The level of intracellular ROS in Huh7 cells was measured by the ROS Detection Assay Kit, following the manufacturer's instructions (BioVision, USA). Briefly, 10^4^ Huh7 cells were seeded in 96-well black bottom plates. The following day, media was removed and cells were washed with 100 μl of ROS Assay Buffer. 100 μl of 1× ROS Label was added, and cells were incubated for 45 min at 37°C in the dark. Next, the ROS label was removed, cells were treated with different doses of ZnO(NP), ZnO(TP), and ZnSO_4_ for 4 h, followed by measurement of fluorescence intensity at excitation and emission wavelengths of 485 and 528 nm, respectively, using a multimode reader (BioTek, USA).

### Cell Cycle Analysis

A total of 0.5 × 10^6^ Huh7 cells were plated in a 12-well plate; the following day, cells were treated with 200 and 400 μM of ZnO(NP), ZnO(TP), and ZnSO_4_ for 24 h, followed by trypsinization and centrifugation at 1,000 × *g* at 4°C for 5 min. Furthermore, the cells were washed twice with ice-cold FACS buffer [PBS (pH 7.4), 2% FBS, 1 mM EDTA], fixed with 70% ice-cold ethanol and incubated for 2 h on ice, resuspended in FACS buffer containing RNAseA, incubated at 37°C for 30 min, and stained with 1 mg/ml propidium iodide (PI) solution for 30 min. In total, 10,000 cells/samples were analyzed by flow cytometer (BD Biosciences, USA), and the data were analyzed using Flowjo software (BD Biosciences, USA).

### HEV Replicon, Electroporation, Infection, Immunofluorescence Assay, and Quantitative Real-Time PCR

Hepatitis E virus infection study was performed in the ORF4-Huh7 cell line as described previously (Kaushik et al., 2017). Cells were maintained in Dulbecco's modified eagle medium (DMEM) containing 10% fetal calf serum (FCS) in a 5% CO_2_ incubator in the presence of 400 μg/ml of hygromycin. Briefly, 4 × 10^5^ cells were plated in a 6-well plate in DMEM + 10% FCS medium. The following day, 8 × 10^6^ genome copies of the virus g1-HEV clinical isolate (Kaushik et al., 2017) were added to the cells, the plates were incubated for 2 h in the CO_2_ incubator, followed by the removal of the infection medium and the addition of fresh medium. After 24 h, the infected cells were treated with 100 and 200 μM of ZnO(NP), ZnO(TP), and ZnSO_4_ for 24 h. After this, total RNA was isolated using TRI reagent and cDNA was synthesized using SuperScript III Reverse Transcriptase (Thermo Fisher Scientific, USA), followed by the quantitative real-time PCR (qRT-PCR). Levels of HEV sense, antisense, and human GAPDH (glyceraldehyde 3-phosphate dehydrogenase) RNA were measured using specific primers, as described earlier (Kaushik et al., 2017). The HEV sense/antisense RNA values were normalized to that of the GAPDH level and represented as the mean ± SEM of three independent experiments, assayed in triplicate.

The P6 HEV-Luc or P6 HEV genomic RNA was synthesized *in vitro* using *Mlu I* linearized pSK P6-Luc or P6-HEV (GenBank accession no. JQ679013.1, g3-HEV) DNA as a template, as described in the previous study (Shukla et al., 2012). Huh7 human hepatoma cells were electroporated with P6 HEV-Luc or P6 HEV RNA. Gaussia-Luciferase activity was measured in the culture medium using the Renilla-Luciferase assay kit, following the manufacturer's instructions (Promega, USA). The viability of the same cells was measured as described above. Gaussia-Luciferase values were normalized to that of cell viability and plotted. The values were represented as the mean ± SEM of three independent experiments performed in triplicate.

For the immunofluorescence assay, 5 days after electroporation, P6-HEV-electroporated cells were grown on coverslips for 24 h, treated with the indicated compounds for 24 h, followed by the staining with anti-ORF2 antibody, as described in the previous study (Kaushik et al., 2017). Anti-rabbit Alexa fluor-488 was used as secondary antibody, and nucleus was stained with DAPI (4′,6-diamidino-2-phenylindole).

For quantitative qRT-PCR assay, 5 days after electroporation, P6-HEV-electroporated cells were grown in 6-well plates for 24 h, treated with the indicated compounds for 24 h in triplicate, followed by the isolation of total RNA and qRT-PCR of P6 HEV RNA level using the following primers: p6-HEV sense forward primer (FP): 5′-ATGCGAATTCCGCGCCGTTGTAACCT and reverse primer (RP): 5′-ACCGGGACAGCGTGGA (Anang et al., 2018). P6-HEV RNA values were normalized to that of the GAPDH level and represented as the mean ± SEM of three independent experiments performed in triplicate.

### HCV Replicon Assay

In order to evaluate the HCV replication, Huh 7.5 cells were transfected with HCV genotype 3a replicon RNA (Madhvi et al., 2017). The HCV-3a replicon expresses a chimeric fusion protein of firefly luciferase and neomycin phosphotransferase and therefore could be selected using G418. The G418-resistant colonies show luciferase activity in proportion to the HCV RNA replication (Madhvi et al., 2017). The G418-resistant replicon expressing Huh 7.5 cells were treated with zinc compounds for 24 h, and luciferase activity was measured using one Glo Luciferase Assay reagents (Promega, USA), following the manufacturer's instructions. Luminescence was detected using a Synergy HT Multi-Mode Microplate Reader (BioTek, USA). The total protein concentration was estimated using Bradford reagent (Bio Rad, USA). Luciferase activity was normalized to that of the total protein amount, and data were represented as the mean ± SEM of three independent experiments performed in triplicate.

### Statistics

Data are presented as means ± SEM of three independent experiments (each experimental sample assayed in triplicate) and were analyzed using GraphPad Prism and a Student's *t*-test. A *P*-value of <0.05 was considered significant.

## Results

### Synthesis of ZnO(NP) and ZnO(TP) and Comparison of Their Cytotoxicity to That Induced by ZnSO_4_

**Figure 1:**
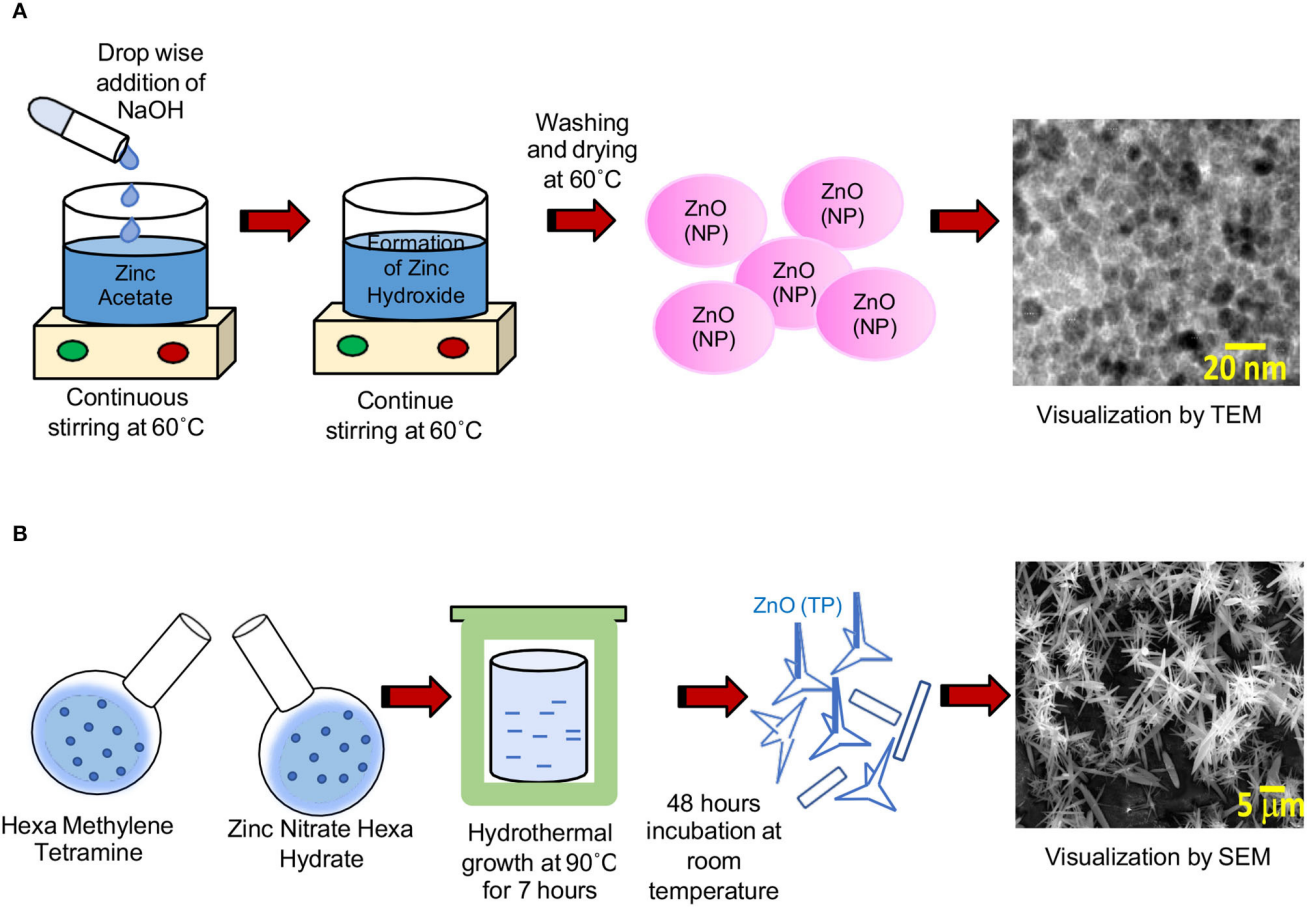
Electron micrograph of ZnO(NP) and ZnO(TP). **(A)** TEM image of ZnO(NP). Scale bar: 20 nm. **(B)** SEM image of ZnO(TP). Scale bar: 5 μm.

Two different ZnO morphologies, namely, nanoparticles (NP) and tetrapods (TP), were synthesized using the chemical route. For the synthesis of ZnO nanoparticles, 30 mM NaOH methanol solution was added to a 10 mM methanol solution of zinc acetate at a volume ratio of 2:1 accompanied by a continuous stirring at 60°C (Ramgir et al., 2020, 2021). The interaction of NaOH and ZnC_4_H_6_O_4_ results in the formation of sodium acetate and Zn(OH)_2_, which subsequently converted to ZnO. The initial turbid or milky solution was stirred for over a 2-h period and the resulting clear solution contains NPs having a size in the range of 5–50 nm. The size of the nanoparticles was measured by transmission electron microscopy (TEM), using a FEI-Titan microscope with an accelerating voltage of 300 kV (Figure 1A). ZnO(TP) was synthesized using the hydrothermal method. A mixture of an aqueous equimolar (25 mM) solution of zinc nitrate hexahydrate and hexamine (hexa methylene tetra amine) was subjected to hydrothermal growth at 90°C for a 7-h period. The solution was further incubated for an additional period of 48 h at room temperature, leading to the formation of a mixed morphology comprising of multi-tetrapods and nanowires. The structure of the ZnO(TP) was visualized by scanning electron microscopy [VEGA Tescan microscope (Figure 1B)]. Notably, ZnO(NP) was detected as round-shaped particles with a size ranging between 5 and 50 nm (Figure 1A). ZnO(TP) exhibited a mixed population of multi-tetrapods and nanowires of ~5 μm in size (Figure 1B).

**Figure 2:**
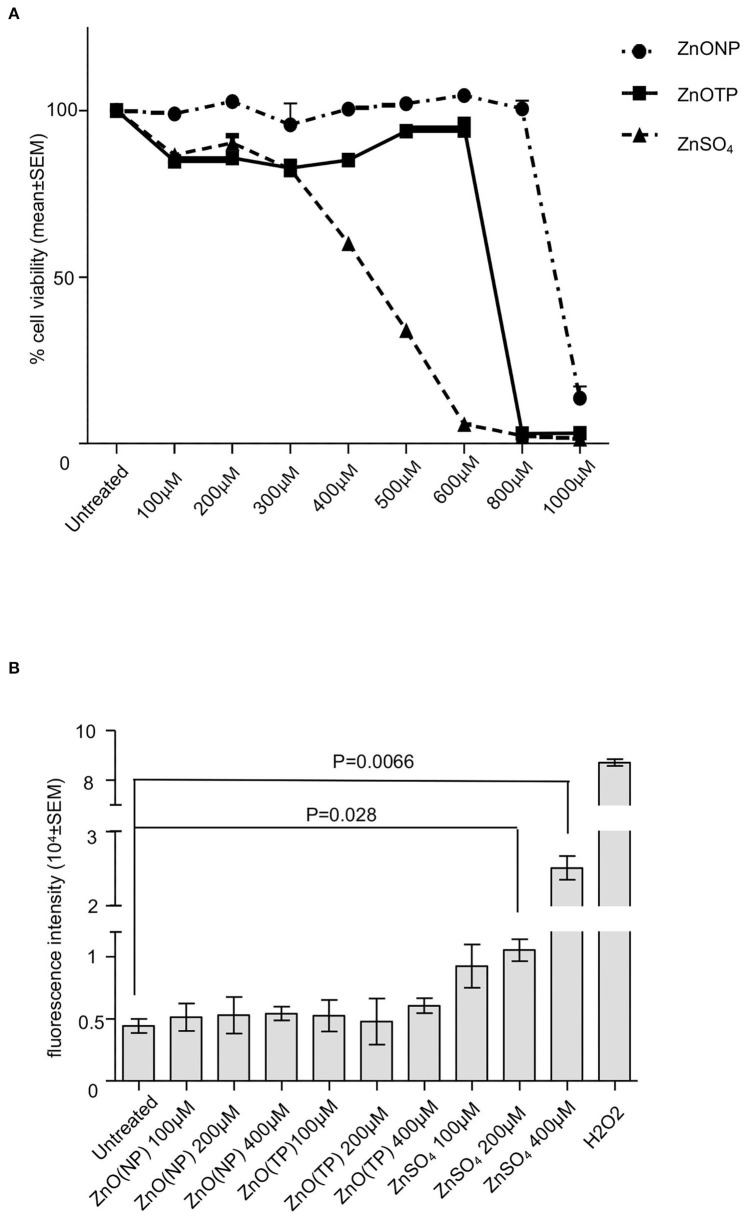
Characterization of ZnO(NP) and ZnO(TP) in mammalian cell culture. **(A)** Percentage viability of Huh7 cells treated for 24 h with increasing dose of ZnO(NP), ZnO(TP), and ZnSO_4_, as indicated. The values for the untreated sample were considered to be 100%, and all other values were calculated with reference to that. Values are mean ± SEM of three independent experiments. **(B)** Estimation of reactive oxygen species (ROS) level in Huh7 cells treated with the different concentration of ZnO(NP), ZnO(TP), and ZnSO_4_ for 6 h. Measured fluorescence intensity was proportional to ROS generation. The mean ± SEM values of triplicate samples are plotted.

Our earlier study has shown that treatment of Huh 7 cells with 500 μM ZnSO_4_ causes 50% cytotoxicity (Kaushik et al., 2017). In order to compare the cytotoxicity of ZnO(NP) and ZnO(TP) to that of ZnSO_4_, Huh7 cells were treated for 24 h with increasing doses of the different compounds, followed by measurement of cell viability. At 900, 700, and 460 μM, 50% toxicity was observed in ZnO(NP), ZnO(TP), and ZnSO_4_ treated cells, respectively (Figure 2A). Next, reactive oxygen species (ROS) levels were measured in ZnO(NP), ZnO(TP), and ZnSO_4_ treated Huh7 cells. ROS was detected using the H_2_DCFDA (2′,7′-dichlorofluorescin diacetate) fluorescence dye. Huh7 cells were treated with 100, 200, and 400 μM of ZnO(NP), ZnO(TP), and ZnSO_4_, respectively, for 6 h, followed by the measurement of fluorescence intensity at excitation and emission wavelengths of 485 and 528 nm, respectively. ZnO(NP) and ZnO(TP) did not significantly alter the ROS level; however, ZnSO_4_ enhanced the ROS levels at 200 and 400 μM (Figure 2B). Hydrogen peroxide (H_2_O_2_) treatment was used as a positive control to monitor the assay conditions, which induced ROS production as expected (Figure 2B).

**Figure 3:**
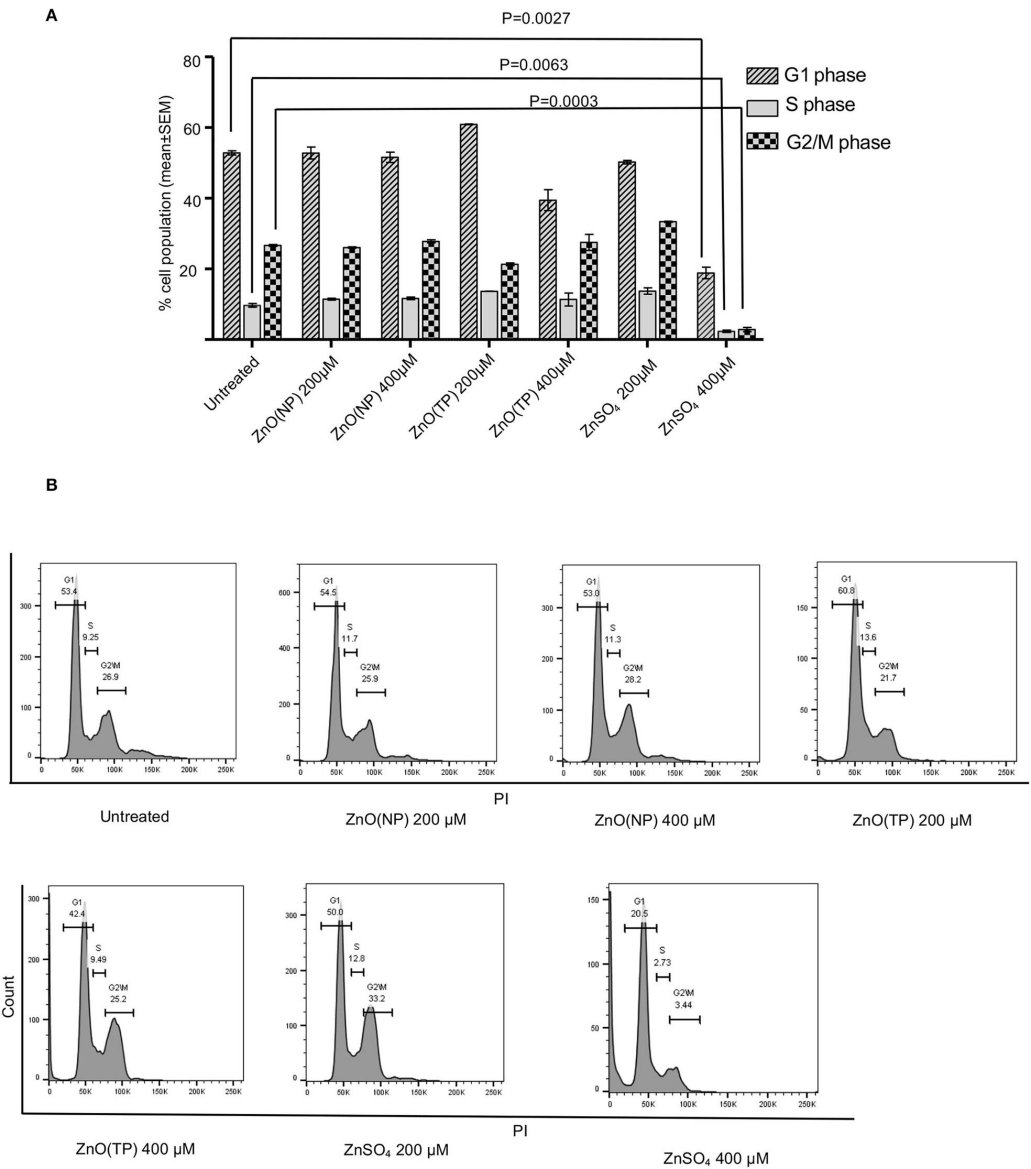
Cell cycle progression analysis after Zinc compound treatment. **(A)** Percentage cell population in G1, S, and G2/M phases of Huh7 cells after 24 h treatment with indicated concentration of ZnO(NP), ZnO(TP), and ZnSO_4_. **(B)** The representative histograms from FACS analysis of percentage of cell population in G1, S, and G2/M phases of Huh7 cells. Values are represented as mean ± SEM of three independent experiments.

Next, the effect of ZnO(NP), ZnO(TP), and ZnSO_4_ on cell-cycle progression was measured. After 24 h of treatment with 200 and 400 μM doses of zinc compounds, different cell-cycle phases were quantified in representative population of Huh7 cells by flowcytometry. Percentage of cells in G1, S, or G2/M phase had no evident changes upon treatment with 200 and 400 μM of ZnO(NP) and ZnO(TP) and 200 μM of ZnSO_4_. However, 400 μM ZnSO_4_ treatment decreased the cell population in all phases, which is in agreement with its observed cytotoxicity (Figures 3A,B). Moreover, there was a significant reduction in the percentage of cells in S and G2/M phases compared to G1 phase upon ZnSO_4_ treatment (Figure 3A).

Collectively, these studies indicated that ZnO(NP) and ZnO(TP) did not induce any cytotoxicity or cytostatic effect at the tested concentrations. Therefore, further studies to evaluate their antiviral properties were conducted within this concentration range.

### Inhibition Studies of ZnO(NP) and ZnO(TP) on HEV Replication

**Figure 4:**
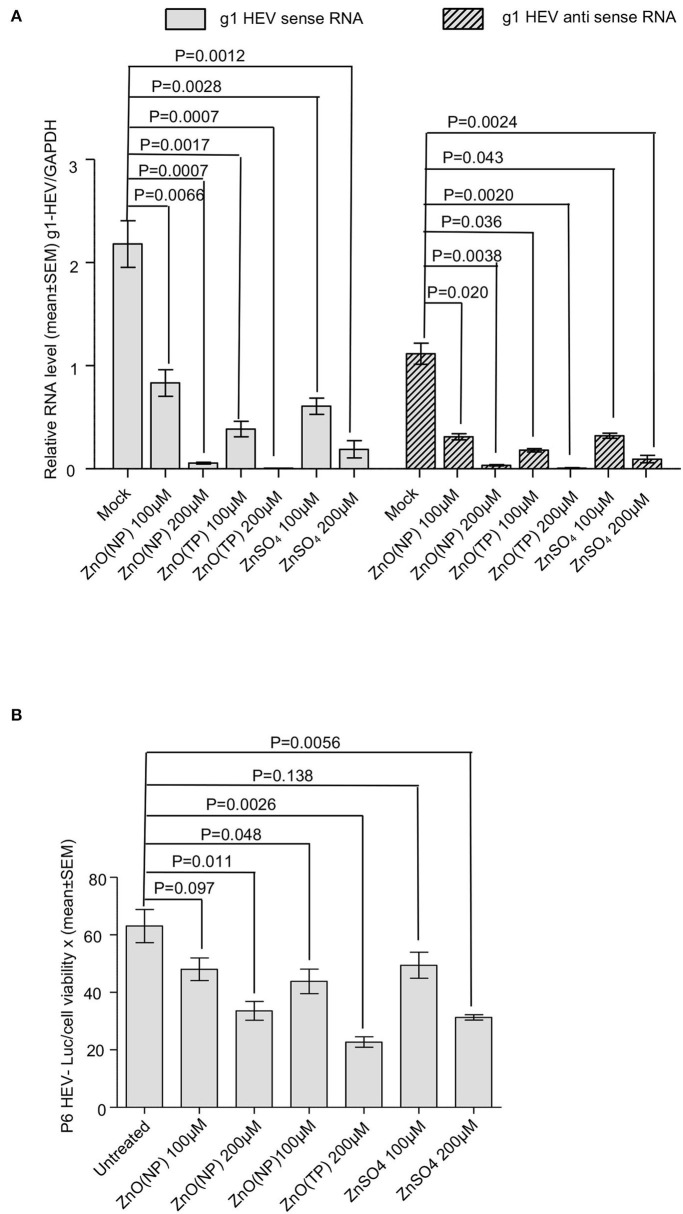
Inhibition of HEV replication by Zinc compounds. **(A)** qRT-PCR detection of the HEV sense and antisense strand RNA levels in ORF4-Huh7 cells infected with a g1-HEV clinical isolate and treated for 24 h with indicated concentration of ZnO(NP), ZnO(TP), and ZnSO_4._ HEV sense/antisense RNA values were normalized to that of the GAPDH and represented as mean ± SEM of three independent experiments. **(B)** Measurement of Gaussia Luciferase (G-Luc) activity in Huh7 cells expressing *in vitro*-synthesized capped RNA of a g3-HEV-Luc replicon and treated for 24 h with indicated concentration of ZnO(NP), ZnO(TP), and ZnSO_4_. G-Luc values were normalized to that of the cell viability and represented as mean ± SEM of three independent experiments. Mock-treated cells were used as a control.

An antiviral effect of zinc compounds on HEV replication was measured using an ORF4-Huh7 cell-based model of g1-HEV infection. We have reported earlier that the Huh7 cells constitutively expressing the ORF4 protein of g1-HEV (ORF4-Huh7 cell line) permit efficient virus replication upon infection with a g1-HEV clinical isolate (Nair et al., 2016). It has been demonstrated that treating these cells with 100 and 200 μM ZnSO_4_ for 24 h significantly inhibit virus replication (Nair et al., 2016). ORF4-Huh7 cells were infected with g1-HEV, treated for 24 h with 100 and 200 μM of ZnO(NP), ZnO(TP), and ZnSO_4_, followed by measurement of g1-HEV sense and antisense RNA levels by quantitative real-time PCR (qRT-PCR). There was a significant reduction in the levels of both sense and antisense RNA in the presence of 100 and 200 μM of ZnO(NP), ZnO(TP), and ZnSO_4_ (Figure 4A). The inhibitory effect of ZnO(TP) was more pronounced than that of ZnO(NP) (Figure 4A).

**Figure 5:**
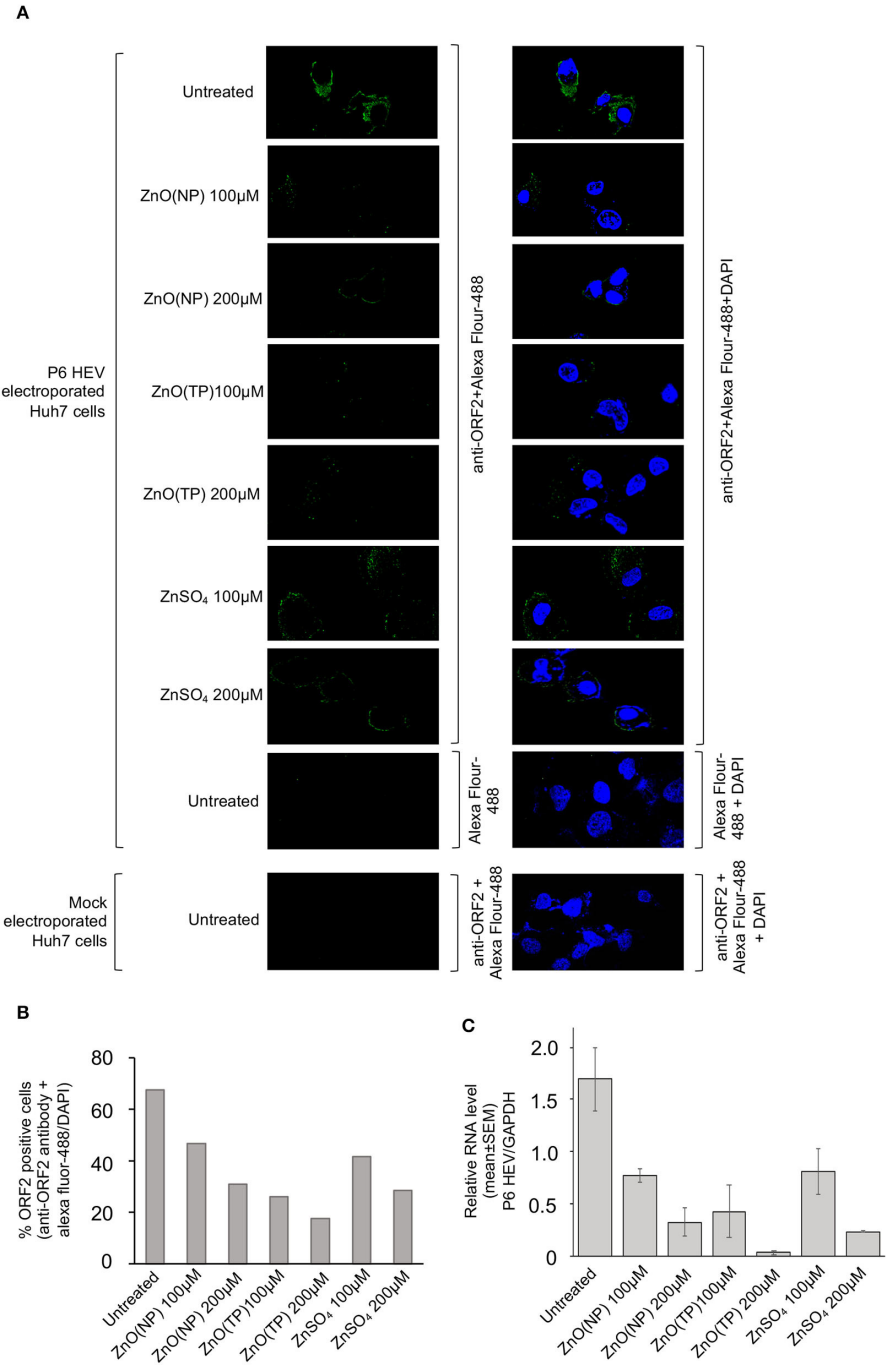
Inhibition of P6 HEV replication by Zinc compounds. **(A)** Immunofluorescence assay-mediated detection of the P6 HEV ORF2 protein level in Huh7 cells electroporated with *in vitro* synthesized capped-genomic RNA of P6 HEV and treated for 24 h with indicated concentration of ZnO(NP), ZnO(TP), and ZnSO_4_. Green and blue represents ORF2 protein and nucleus, respectively. Magnification: 600×. Left panel shows ORF2 staining (Alexa fluor-488) and right panel shows superimposed image of ORF2 (Alexa fluor-488) and nucleus (DAPI) staining. **(B)** Graphical representation of average of % ORF2 positive cells estimated from ten random fields of samples shown in **(A)**. **(C)** qRT-PCR detection of the P6 HEV genomic RNA level in Huh7 cells electroporated with *in vitro* synthesized capped-genomic RNA of P6 HEV and treated for 24 h with indicated concentration of ZnO(NP), ZnO(TP), and ZnSO_4_. P6 HEV RNA values were normalized to that of the GAPDH and represented as mean ± SEM of three independent experiments.

Next, a Huh7 cell-based model of g3-HEV replicon expressing Gaussia-luciferase (P6 HEV-Luc) was used to estimate the effect of zinc compounds on the replication of g3-HEV. Huh7 cells expressing *in vitro*-synthesized capped genomic RNA of P6 HEV-Luc replicon were treated with 100 and 200 μM of ZnO(NP), ZnO(TP), or ZnSO_4_ for 24 h. Luciferase activity was significantly low in cells treated with the zinc compounds, indicating inhibition of viral replication in those cells (Figure 4B). The above results were further confirmed by using a Huh7 cell-based model of infectious g3-HEV (P6 HEV). Huh7 cells were electroporated with capped genomic RNA of P6 HEV, followed by treatment of the cells with different zinc compounds. Immunofluorescent staining of the viral ORF2 protein showed a significant decrease in its level in the presence of different zinc compounds, in agreement with the results obtained in the P6-HEV-Luc-expressing cells and g1-HEV-infected cells (Figure 5A). The percentage of ORF2-positive cells was estimated after counting the number of ORF2-positive cells in 10 randomly selected fields on each slide. The average percentages of the samples are represented in the graph, which shows a dose-dependent reduction in ORF2-positive cells in the presence of all zinc compounds (Figure 5B). Next, parallelly processed samples were analyzed by qRT-PCR to measure the level of P6 HEV genomic RNA. As expected, there was a dose-dependent reduction in P6 HEV genomic RNA level in the presence of all zinc compounds (Figure 5C).

**Figure 6:**
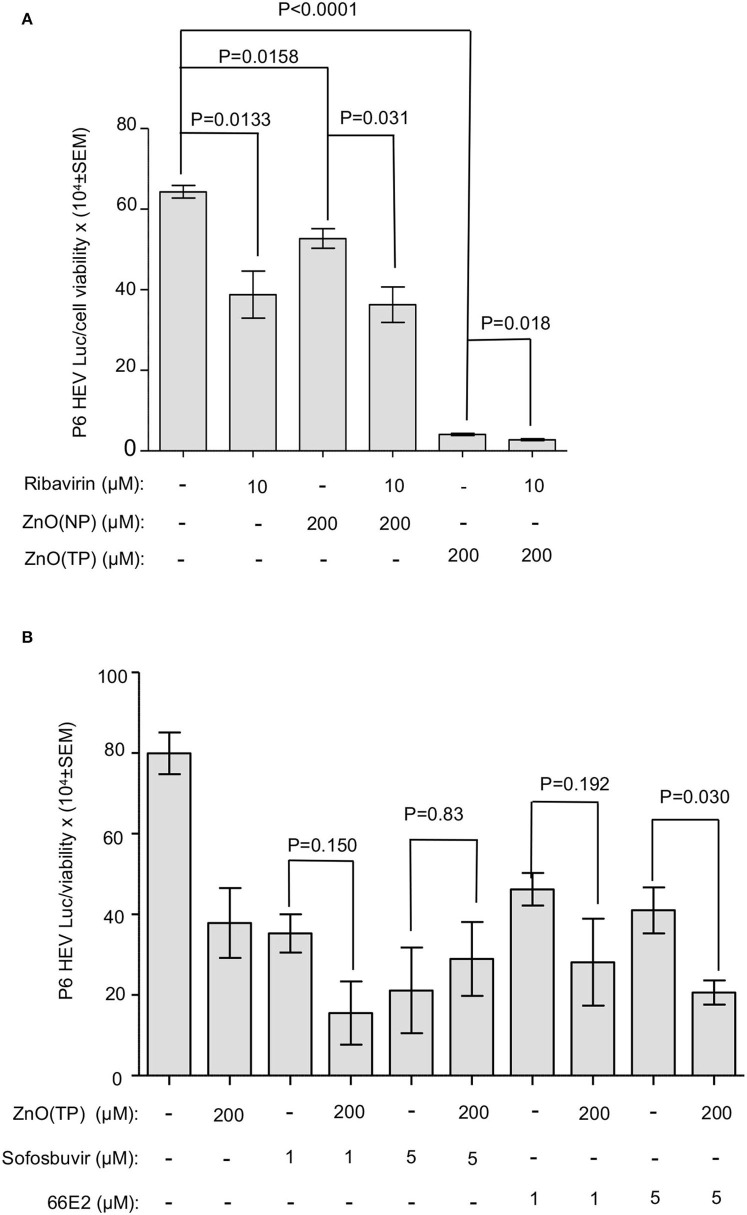
Effect of co-treatment of zinc compounds and known anti-HEV compounds on g3-HEV replication. **(A)** Measurement of G-Luc activity in Huh7 cells expressing *in vitro* synthesized capped RNA of a g3-HEV-Luc replicon and treated for 3 days (once daily) with the indicated concentration of different compounds. G-Luc values were normalized to that of the cell viability and represented as mean ± SEM. **(B)** Measurement of G-Luc activity in Huh7 cells expressing *in vitro* synthesized capped RNA of a g3-HEV-Luc replicon and treated for 24 h with the indicated concentration of different compounds. G-Luc values were normalized to that of the cell viability and represented as mean ± SEM of three independent experiments.

Ribavirin, 66E2, and sofosbuvir have been shown to inhibit HEV replication (Thi et al., 2016; Madhvi et al., 2017). The effect of cotreatment of ZnO(NP) or ZnO(TP) and known antivirals on HEV replication have been checked. A Huh7 cell-based model of the P6 HEV-Luc replicon was used for the above study. P6 HEV-Luc replicon-expressing cells were treated with 10 μM of ribavirin along with 200 μM of ZnO(NP), ZnO(TP), or ZnSO_4_ for 72 h (treatment repeated at 24-h intervals). Ribavirin-treated cells were maintained in parallel as a control. As expected, treatment with ribavirin, ZnO(NP) or ZnO(TP) significantly inhibited viral replication (Figure 6A). Interestingly, we also observed that ZnO(TP) was much more efficient than ribavirin and ZnO(NP) in inhibiting viral replication after 3 days of treatment. Cotreatment with ribavirin and ZnO(NP) or ZnO(TP) marginally enhanced the inhibitory effect (Figure 6A). Next, P6 HEV-Luc replicon-expressing cells were treated for 24 h with sofosbuvir and 66E2, followed by measurement of Luciferase levels. As expected, both sofosbuvir and 66E2 inhibited HEV replication (Figure 6B). There was no cooperative or antagonistic effect of sofosbuvir and ZnO(TP) or 66E2 and ZnO(TP) cotreatment on HEV replication (Figure 6B).

### Inhibition Studies of HCV Replication by ZnO(NP) and ZnO(TP)

**Figure 7:**
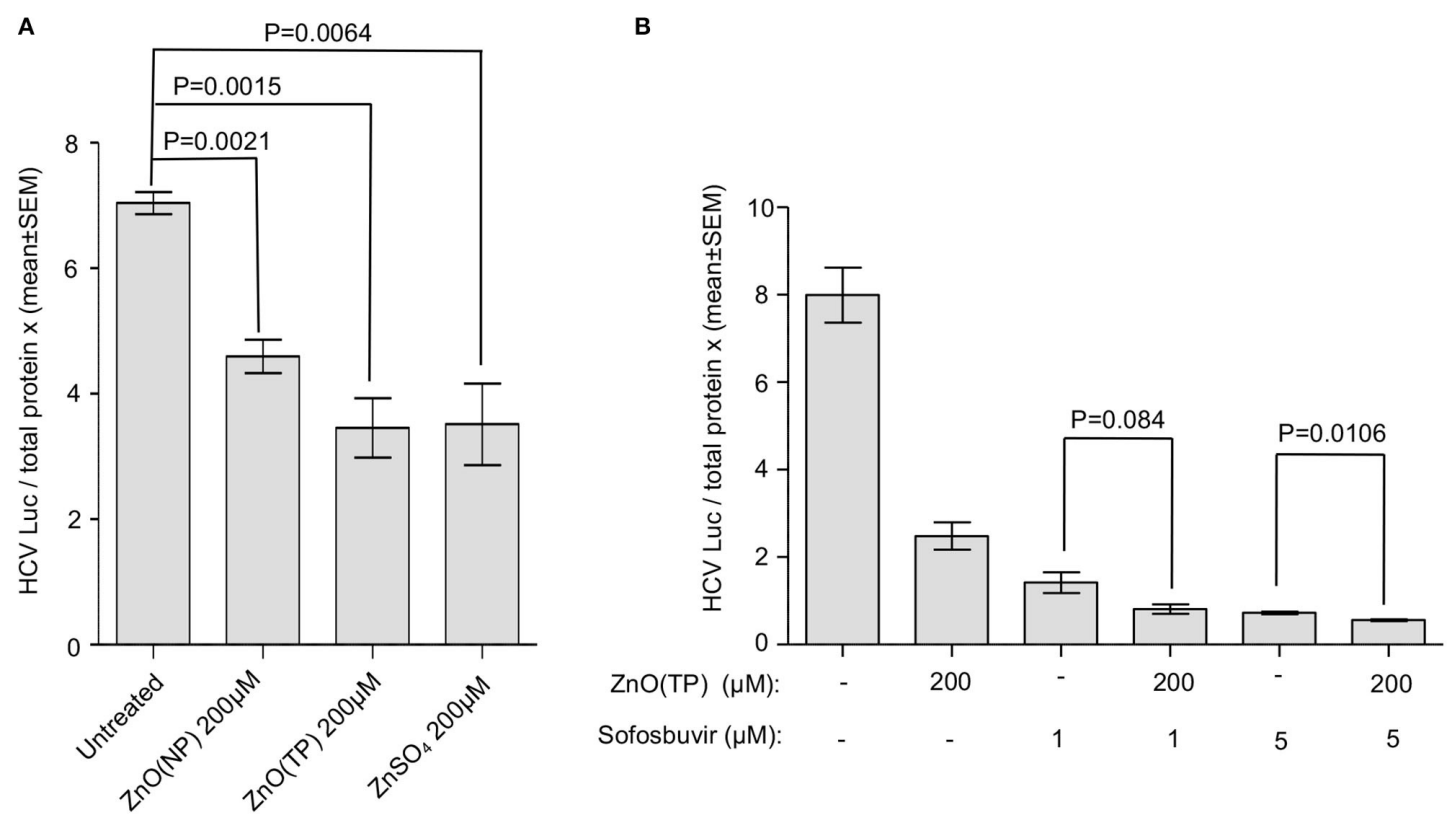
Inhibition of HCV replication by zinc compounds. **(A)** G418-resistant g3a-HCV replicon expressing Huh7.5 cells were treated with indicated doses of ZnO(NP), ZnO(TP), and ZnSO_4_ compounds for 24 h. Firefly luciferase activity and total protein concentration were measured. Luciferase/total protein ratio is represented in the graph as mean ± SEM of three independent experiments. **(B)** G418-resistant g3a-HCV replicon expressing Huh7.5 cells were treated with 1 and 5 μM sofosbuvir alone or cotreated with 200 μM ZnO(NP) and ZnO(TP), respectively, for 24 h. Luciferase/total protein ratio is represented in the graph as mean ± SEM of three independent experiments.

Yuasa et al. (2006) have reported that zinc salts inhibit HCV replication *in vitro*. Therefore, we tested the effect of ZnO(NP) and ZnO(TP) on HCV replication. Huh7.5 cells-expressing HCV-3a replicon was treated for 24 h with 200 μM of ZnO(NP), ZnO(TP), or ZnSO_4_, and the firefly luciferase activity was measured. An aliquot of the same cell lysate was used to quantify the level of total protein that was used to normalize the luciferase activity values. As expected, ZnSO_4_ treatment significantly inhibited HCV replication (Figure 7A). Both ZnO(NP) and ZnO(TP) treatments also inhibited HCV replication (Figure 7A). Since sofosbuvir is a well-known direct-acting antiviral against HCV, the effect of cotreatment of sofosbuvir and ZnO(TP) using the abovementioned Huh7.5 cell-based replicon model of HCV-3a was tested. As expected, both sofosbuvir and ZnO(TP) significantly inhibited HCV replication at the tested concentrations. However, no synergistic or antagonistic effect was observed in cells cotreated with sofosbuvir and ZnO(TP) (Figure 7B).

## Discussion

In this study, the cytotoxic and cytostatic effects of nanoparticles and tetrapods of ZnO have been extensively evaluated, and their potential as antivirals against HEV and HCV has been established successfully. Our results clearly demonstrate the potent inhibitory effect of ZnO(NP) and ZnO(TP) on g1-HEV, g3-HEV, and g3A-HCV. Both the morphologies of ZnO were found to be safer than the conventional zinc salt, namely, ZnSO_4_. One of the major limitations of therapeutic use of zinc is attributed to toxicity associated with high zinc doses, as required in zinc supplementation therapy. Excess zinc is toxic to the cell as it initiates intracellular signaling pathways. This eventually leads to the production of different types of ROS, which apparently damage lipids, proteins, carbohydrates, and DNA and eventually causes cell death (Hamatake et al., 2000; Plum et al., 2010). Therefore, the lower toxicity of ZnO(NP) and ZnO(TP) while retaining potent antiviral activity supports their therapeutic value. The therapy has advantages such as enhanced efficacy, reduced side effects, and more targeted localization of disease sites. Unique three-dimensional structures and surface characteristics are the likely contributors to nanoparticle efficacy and toxicity (Nie et al., 2006; Yuasa et al., 2006; De Jong and Borm, 2008; Mecklenburg et al., 2012; Papavlassopoulos et al., 2014; Modi, 2015; Sirelkhatim et al., 2015; Antoine et al., 2016).

Our study shows ZnO(TP) to be more effective as an antiviral compared to ZnO(NP). As seen in the electron micrograph, ZnO(TP) displays an entirely different structure than ZnO(NP). It is visible as a multitetrapod of nanorods/nanowires. It is known that ZnO has more than one stable structure, a property usually referred to as polytypism. Importantly, it can be found in hexagonal as well as cubic structures, i.e., wurtzite and/or zinc blend structures. In the case of a multipod or tetrapod structure, the core tetrapod is usually formed of a zinc blend structure while the arms are a hexagonal wurtzite structure. Herein, the (0001) facets of wurtzite and (111) of the zinc blend serve the purpose of intermediate facets for the pod type morphology. This unique morphology might be contributing to the superior antiviral effect of ZnO(TP). ZnO(TP) has been shown to suppress HSV infection by binding and entrapping the virus, which prevents viral entry into the host cell. Our earlier study has shown that zinc salts do not affect HEV entry. They inhibit HEV infection by interfering with viral replication. ZnO(NP) and ZnO(TP) inhibited infection by g1-HEV clinical isolates, which rely on the cellular entry step. ZnO(NP) and ZnO(TP) also inhibited replication of g3-HEV and g3a-HCV replicons, which do not rely on a cellular entry step. These results suggest that ZnO(NP) and ZnO(TP) execute their antiviral function independent of the viral entry step, likely by inhibiting the viral replication step. Future studies should aim at further molecular characterization of the antiviral function and evaluation of the potential therapeutic benefit of ZnO(NP) and ZnO(TP) in the management of HEV- and HCV-induced hepatitis.

## Conclusion

The cytotoxic and cytostatic effects of nanoparticles and tetrapods of ZnO have been extensively evaluated, and their potential as antivirals against HEV and HCV has been established successfully. Our results clearly demonstrate the potent inhibitory effect of ZnO(NP) and ZnO(TP) on g1-HEV, g3-HEV, and g3A-HCV. Both the morphologies of ZnO were found to be safer than the conventional zinc salt, namely, ZnSO_4_. Besides, the lower toxicity of ZnO(NP) and ZnO(TP) while retaining potent antiviral activity supports their therapeutic value. Importantly, ZnO(TP) was found to be more effective as an antiviral compared to ZnO(NP). It suppresses HSV infection by binding and entrapping the virus, thereby preventing its entry into the host cell. Both the morphologies also inhibited infection by g1-HEV clinical isolate and replication of g3-HEV and g3a-HCV replicons. Most importantly, the antiviral function is found to be independent of the viral entry step and is governed by the inhibition of the viral replication step. Thus, our results clearly demonstrate the potential of ZnO nanoparticles and tetrapods toward management of HEV and HCV induced hepatitis.

## Data Availability Statement

The raw data supporting the conclusions of this article will be made available by the authors, without undue reservation.

## Author Contributions

JGu and MS performed experimental design and data analysis. JGu, MS, and NR wrote the manuscript. JGa, MI, SA, and NR performed experiments. AD, KM, and CR-K contributed reagents. All authors edited the manuscript.

## Funding

This study was supported by a grant to MS by the Indian Council of Medical Research (ICMR), Government of India. Part of the study was supported by intramural FRGS grant of GGSIPU to CR-K. JGu is supported by a post-doctoral fellowship from ICMR, and JGa is supported by a post-doctoral fellowship from the Department of Biotechnology, Government of India.

## Conflict of Interest

The authors declare that the research was conducted in the absence of any commercial or financial relationships that could be construed as a potential conflict of interest.

## Publisher's Note

All claims expressed in this article are solely those of the authors and do not necessarily represent those of their affiliated organizations, or those of the publisher, the editors and the reviewers. Any product that may be evaluated in this article, or claim that may be made by its manufacturer, is not guaranteed or endorsed by the publisher.
